# Atherogenic index of plasma (AIP): a novel predictive indicator for the coronary artery disease in postmenopausal women

**DOI:** 10.1186/s12944-018-0828-z

**Published:** 2018-08-22

**Authors:** Ting-Ting Wu, Ying Gao, Ying-Ying Zheng, Yi-Tong Ma, Xiang Xie

**Affiliations:** 1 Department of Cardiology, First Clinical Medical College & First Affiliated Hospital of Xinjiang Medical University, Urumqi, 830054 People’s Republic of China; 2 Cadre Ward, First Clinical Medical College & First Affiliated Hospital of Xinjiang Medical University, Urumqi, 830054 People’s Republic of China; 3 Department of Cardiology, First Affiliated Hospital of Zhengzhou University, Zhengzhou, 450000 People’s Republic of China

**Keywords:** Atherogenic index of plasma, Coronary artery disease, Postmenopausal women, Lipids

## Abstract

**Background:**

Dyslipidemia is one of the most important factors for coronary artery disease (CAD). Atherogenic index of plasma (AIP) is a novel indicator involved in dyslipidemia. However, the relation between AIP and CAD in postmenopausal women remains unclear. We hypotheses that AIP is a strong predictive indicator of CAD in postmenopausal women.

**Methods:**

A propensity score matching case–control study including 348 postmenopausal CAD cases and 348 controls was conducted in the present study.

**Results:**

Compared with controls, CAD patients had higher levels of total cholesterol (TC), triglyceride (TG), low-density lipoprotein cholesterol (LDL-C) and apolipoprotein B (APOB), but lower high-density lipoprotein cholesterol (HDL-C) and apolipoprotein A-1 (APOA-1). The values of nontraditional lipid profiles, including non-HDL-C, TC/HDL-C, LDL-C/HDL-C, non-HDL-C/HDL-C (atherogenic index, AI), TC∗TG∗LDL/HDL-C (lipoprotein combine index, LCI), log(TG/HDL-C) (atherogenic index of plasma, AIP) and APOB/APOA-1 were all significantly higher in the CAD patients. The results of Pearson correlation analyses showed AIP was positively and significantly correlated with TC (*r* = 0.092, *P* < 0.001), TG (*r* = 0.775, *P* = 0.015), APOB (*r* = 0.140, *P* < 0.001), non-HDL-C (*r* = 0.295, *P* < 0.001), TC/HDL-C (*r* = 0.626, *P* < 0.001), LDL-C/HDL-C (*r* = 0.469, *P* < 0.001), AI (*r* = 0.626, *P* < 0.001), LCI (*r* = 0.665, *P* < 0.001), APOB/APOA-1(*r* = 0.290, *P* < 0.001) and was negatively correlated with APOA-1 (*r* = − 0.278, *P* < 0.001) and HDL-C (*r* = − 0.665, *P* < 0.001). In the multivariate logistic regression analysis, AIP was an independent predictor of CAD. After adjusting for the traditional clinical prognostic factors including diabetes and hypertension, we found AIP could be an independent risk factor for CAD (odds ratio [OR], 3.290; 95% confidence interval [CI], 1.842–5.877, *P* < 0.001). After adjusting for multiple clinical factors include diabetes, hypertension, smoking, heart ratio, fasting blood glucose, we found AIP also could a powerful risk factor, OR = 3.619, 95%CI (2.003–6.538), *P* < 0.001.

**Conclusion:**

The present study indicated that AIP might be a strong marker for predicting the risk of CAD in postmenopausal women.

## Background

The ability of plasma lipid to migrate into the subintimal layer is an important step to developing atherosclerosis. Lipid and its lipoprotein constituent have been designated as a mediator and a marker of coronary heart disease (CAD). It is characterized by high ratio of low-density lipoprotein cholesterol (LDL-C) to high-density lipoprotein cholesterol (HDL-C) and increased level of triglycerides (TG) [1]. The association between lipoprotein concentration and cardiovascular disease (CVD) risk has been studied by numerous authors for decades, and total cholesterol (TC) and LDL-C are well known cardiovascular risk factors. Elevated TG and a low HDL level are strong markers of cardiovascular diseases. An increase of TG levels causes an increase in the small dense LDL (sdLDL) level and finally causes an increase of cardiovascular risk [2–5]. Recently, it was determined that the atherogenic index of plasma (AIP) value, which is acquired by the logarithmic transformation of the number found by dividing plasma TG value to HDL value, can be a good marker for the risk of atherosclerosis and cardiovascular disease [6–8]. It was reported that an AI*P* value of 0.1–0.24 shows medium cardiac risk [9].

Menopause, which is the permanent cessation of menstruation following loss of ovarian activity, has considerable impact on social, reproductive, physical and psychological health of the woman. It has been shown that postmenopausal women have less cardiovascular friendly lipid profiles than before menopause [10]. Data from cross-sectional and longitudinal studies have shown that menopause alters CVD risk factors. Postmenopausal compared with premenopausal women have higher plasma TC, LDL-C, very low-density lipoprotein cholesterol (VLDL-C), and TG levels [11].There is convincing evidence that menopause is associated with central adiposity [12], increased diastolic blood pressure [13] and increased insulin resistance [14], hence an increased likelihood to develop CVD. Thus, the true value of the AIP is yet to be determined in postmenopausal women. To this end, we undertook the present study, which aimed at investigating the link between AIP and risk of CAD and finding out whether AIP might be a better marker for predicting the risk of CAD among postmenopausal women.

## Methods

### Study population

**Fig. 1:**
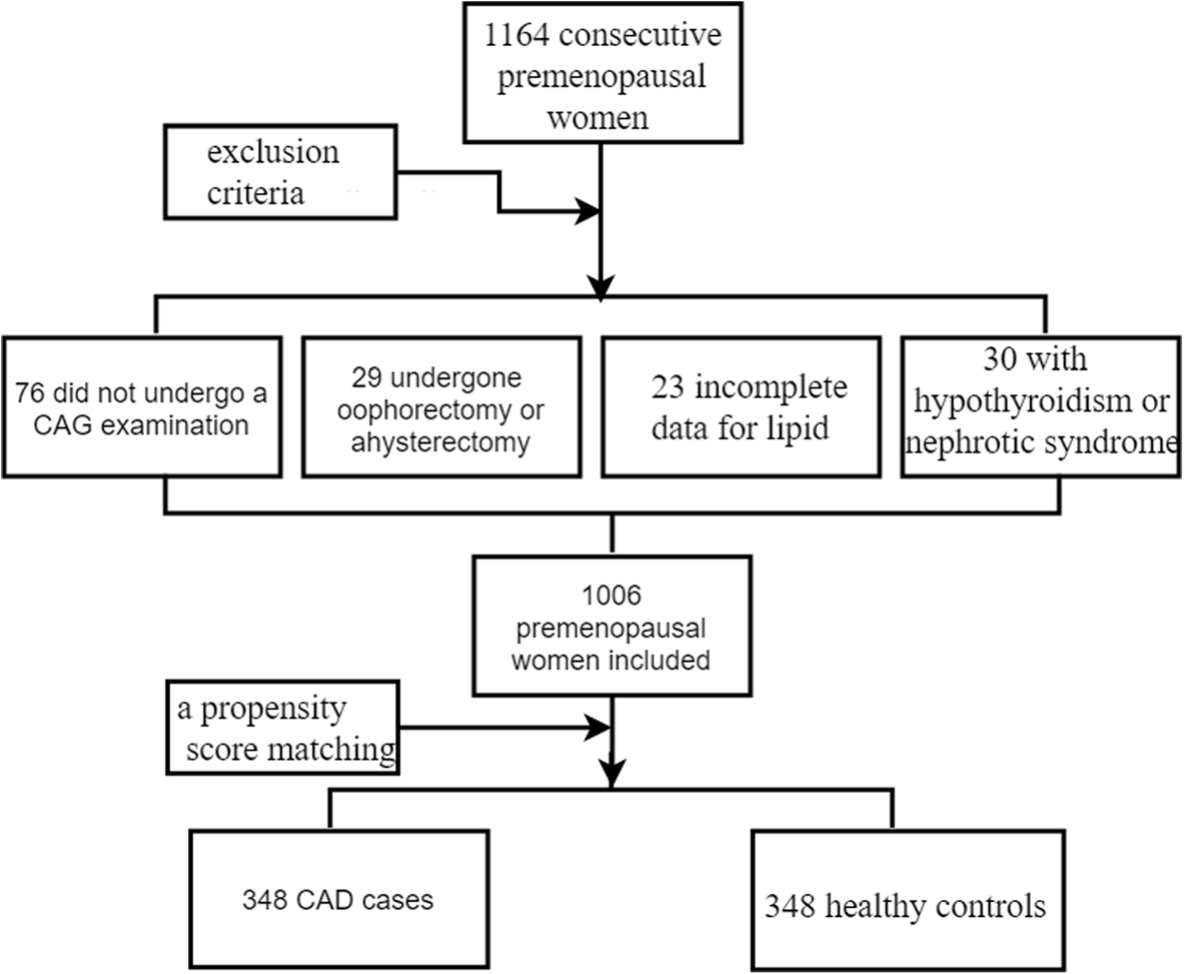
The flow chart of the study

The exclusion criteria were the following: patients who had undergone a bilateral oophorectomy or a hysterectomy, with or without ovarian preservation; patients who did not undergo a coronary artery angiography (CAG) examination; incomplete data for traditional lipid profiles; and patients with hypothyroidism or nephrotic syndrome. According to the exclusion criteria, 1006 consecutive premenopausal women with 600 CAD patients, 406 healthy controls were enrolled in this study between January 2012 and January 2015 who were hospitalized in the First Affiliated Hospital of Xinjiang Medical University. After a propensity score matching for age, a total of 348 CAD cases and 348 apparently healthy premenopausal women served as control subjects were enrolled in this study. The study protocol was approved by the Ethics Committee of the First Affiliated Hospital of Xinjiang Medical University. A flowchart outlining our study was shown in Fig. 1.

### Definition

Essential hypertension(EH) was defined as a systolic blood pressure of ≥140 mmHg and/or a diastolic blood pressure of ≥90 mmHg in at least 2 measurements or use of any antihypertensive drug. Diabetes mellitus (DM) was defined as fasting plasma glucose levels 126 mg/dl on multiple measurements or current use of anti-diabetic medications. Smoking status was defined as current tobacco use. Menopause was defined as amenorrhoea for at least 12 months and elevated gonadotrophin levels (FSH > 40 IU/l).CAD was defined in accordance with the 1979 WHO diagnostic criteria [15], and all of the patients underwent a CAG examination. The CAG examinations were performed using Judkin technique via the radial or femoral artery. Angiograms were analyzed by 2 experienced doctors who were blinded to this study. Control subjects were defined as those lacking typical angina pectoris symptoms and those in whom stenosis of the major coronary arteries was less than 50%.Scoring of CAD severity was performed with a modification of the coronary atherosclerosis scoring system named the standard of Gensini method, as described by Gensini GG [16] . The Gensini score is an anatomic scoring system and quantifies the properties of lesions including their complexity, morphology, and locations in the coronary tree. Gensini score was used to calculate the levels of coronary severity scores (CSS).

The AIP is defined as the base 10 logarithm of the ratio of the concentration of TG to HDL-C, where each concentration is expressed in mmol/L; The non-HDL-C is defined as TC minus HDL-C; The atherogenic index (AI) is defined as the ratio of non-HDL-C to HDL-C; The lipoprotein combine index(LCI) is defined as the ratio of TC∗TG∗LDL to HDL-C.

### Laboratory assessments

Peripheral venous blood samples of the patients were obtained on admission in the inpatient ward. Data regarding clinical and demographic characteristics including age, history of hypertension and diabetes mellitus and smoking status were collected from medical records. Laboratory data including urea nitrogen, creatinine, uric acid, fasting blood glucose, bilirubin and lipid parameters as TC, TG, HDL-C, LDL-C, APOA-1, APOB were noted.

### Statistical analysis

Statistical analyses were carried out in SPSS 22.0 Statistical Package Program for Windows (SPSS Inc., Chicago, Illinois). The non-HDL-C, TC/HDL-C, LDL-C/HDL-C, LCI, AI, AIP and APOB/APOA-1concentration ratios were calculated. Continuous variables were presented as the mean ± standard deviation (SD) and were compared using an independent samples t test. However, if the data were not accorded with normal distribution, nonparametric test should be used. Categorical variables were expressed as frequencies and percentages and were compared using Chi-square tests. The correlation between the AIP values and other variables were calculated by Pearson correlation analysis. Both univariate and multivariate logistic regression analyses were performed to explore the relationship between the lipid parameters and risk of CAD. The adjusted odds ratio (OR) per 1 SD increase in the corresponding lipid variable and 95% confidence intervals (95%CIs) were calculated. A value of *P* < 0.05 in a 2-sided test was considered significant.

## Results

### Basic characteristics and cardiovascular risk factors of subjects

After a propensity score matching for age, a case–control study includes a total of 696 postmenopausal women were enrolled in this study, and the mean age of subjects is 61.7 ± 7.1.We can see that subjects diagnosed with CAD were more likely to have higher known history of hypertension, diabetes mellitus and smoking. Higher heart rate, fasting blood glucose and higher traditional lipid parameters as TG, TC, LDL-C, APOB, and lower HDL-C, APOA-1.The median of LCI was16.27, and after a normal test being carried out, a nonparametric test was used in LCI (*P* < 0.001). The nontraditional lipid profiles were much significantly higher in CAD cases (*P* < 0.05). The distribution of various clinical and biological variables measured is depicted by Table 1**.**

**Table 1:** Baseline characteristics of the both CAD and control group

Variables	CAD cases(*n* = 348)	Controls(n = 348)	*t (x* ^*2*^ *)*	*P* value
EH [n(%)]	213(61.2%)	177(50.9%)	7.558	0.006
DM [n(%)]	89(25.6%)	62(17.8%)	6.165	0.013
Smoking [n(%)]	67(19.5%)	43(12.4%)	6.478	0.011
Heart Rate (bpm)	76 ± 11	74 ± 11	0.173	0.031
SBP(mmHg)	130 ± 15	129 ± 9	37.274	0.232
BUN (mmol/L)	5.47 ± 1.59	5.44 ± 1.57	0.039	0.824
Creatinine (mmol/L)	74.06 ± 19.03	72.68 ± 15.60	4.015	0.296
Uric acid (mmol/L)	324.46 ± 85.22	315.24 ± 95.94	1.433	0.181
FBG (mmol/L)	6.32 ± 2.76	5.85 ± 2.21	8.138	0.013
TBIL (mmol/L)	12.83 ± 7.08	12.28 ± 6.28	4.421	0.283
DBIL (mmol/L)	4.22 ± 3.64	3.73 ± 3.34	1.980	0.066
IBIL (mmol/L)	8.67 ± 6.30	8.57 ± 5.13	6.887	0.822
BMI (kg/m^2^)	26.27 ± 3.29	25.84 ± 3.83	6.201	0.111
Traditional lipid profiles				
TG(mmol/l)	1.89 ± 1.34	1.65 ± 0.93	2.239	0.007
TC(mmol/l)	4.56 ± 1.20	4.39 ± 0.98	9.530	0.043
HDL-C(mmol/l)	1.09 ± 0.29	1.20 ± 0. 32	0.812	< 0.0001
LDL-C(mmol/l)	2.82 ± 1.00	2.61 ± 0.85	10.801	0.003
APOA-1(g/l)	1.27 ± 0.25	1.31 ± 0.24	0.619	0.048
APOB(g/l)	0.93 ± 0.31	0.87 ± 0.25	15.384	0.009
Nontraditional lipid profiles				
non-HDL-C	3.47 ± 1.28	3.20 ± 0.94	11.971	0.001
TC/HDL-C	4.43 ± 1.56	3.95 ± 1.79	2.136	< 0.0001
LDL-C/HDL-C	2.72 ± 1.12	2.35 ± 1.16	3.040	< 0.0001
AI	3.43 ± 1.56	2.95 ± 1.79	2.136	< 0.0001
AIP	0.20 ± 0.27	0.10 ± 0.27	0.434	< 0.0001
APOB/APOA-1	0.75 ± 0.26	0.68 ± 0.22	13.345	< 0.0001

*AI* atherosclerosis index, *AIP* atherogenic index of plasma, *CAD* coronary artery disease, *DM* diabetes mellitus, *EH* essential hypertension, *TG* triglyceride, *HDL-C* high-density lipoprotein cholesterol, *LCI* lipoprotein combined index, *LDL-C* low-density lipoprotein cholesterol, *TC* total cholesterol, *BMI* body mass index, *BUN* Blood urea nitrogen, *FBG* fasting blood glucose, *TBIL* total bilirubin, *DBIL* direct bilirubin, *IBIL* indirect bilirubin, *APOA-1* apolipoprotein A-1, *APOB* apolipoprotein B, *APOB/APOA-1* apolipoprotein B to apolipoprotein A-1 ratio

### Correlation between AIP and other parameters

Pearson correlation analyses were performed to investigate the correlation of AIP with other continuous variables. As summarized in Table 2, AIP was positively and significantly correlated with TC (*r* = 0.092, *P* < 0.001), TG (*r* = 0.775, *P* = 0.015), APOB (*r* = 0.140, *P* < 0.001), non-HDL-C (*r* = 0.295, *P* < 0.001), TC/HDL-C (*r* = 0.626, *P* < 0.001), LDL-C/HDL-C (*r* = 0.469, *P* < 0.001), AI (*r* = 0.626, *P* < 0.001),LCI (*r* = 0.665, *P* < 0.001), APOB/APOA-1(*r* = 0.290, *P* < 0.001) and was negatively correlated with APOA-1 (*r* = − 0.278, *P* < 0.001) and HDL-C (*r* = − 0.665, *P* < 0.001).

**Table 2:** Correlation between PAI and other variables

	TC	TG	HDL	LDL	APOA-1	APOB	Non-HDL	LDL/HDL	TC/HDL	LCI	AI	APOB/APOA-1	Gensini
AIP	0.092^*^	0.775^*^	−0.665^*^	0.004	−0.278^*^	0.140^*^	0.295^*^	0.469^*^	0.626^*^	0.665^*^	0.626^*^	0.29^*^	0.133^*^
	− 0.057	0.039	0.053	−0.035	−0.039	− 0.003	−0.010	− 0.038	−0.058	− 0.010	−0.013	0.164^*^	0.087^*^
	Age	BMI	SBP	HR	FBG	BUN	UA	TBIL	DBIL	IBIL	Smoke	DM	EH

*P < 0.05

AIP was also positively and significantly correlated with EH(*r* = 0.087; *p* = 0.021), DM (*r* = 0.164; *p* < 0.001), Gensini score(*r* = 0.133; *p* < 0.001), but not with age (*r* = − 0.057; *p* = 0.135), active smoking (*r* = − 0.013; *p* = 0.742), BMI (*r* = 0.039; *p* = 0.304), SBP (*r* = 0.053;*p* = 0.166),HR (*r* = − 0.035; *p* = 0.363),BUN(*r* = − 0.003; *p* = 0.947), uric acid (*r* = − 0.010; *p* = 0.785), FBG (*r* = − 0.039; *p* = 0.308), CR(*r* = 0.003; *p* = 0.930), TBIL(*r* = − 0.038; *p* = 0.314),DBIL(*r* = − 0.058; *p* = 0.124) nor IBIL(r = − 0.010; *p* = 0.792).

### Univariate and multivariate logistic regression analysis

In univariate logistic regression analysis, we can see all lipid parameters were predictors for CAD risk. To eliminate interference from other confounding factors, multivariate logistic regression analyses were made. In model 1, after adjusting for the traditional clinical prognostic factors including diabetes and hypertension, we found AIP could be an independent risk factor for CAD (odds ratio [OR], 3.290; 95% confidence interval [CI], 1.842–5.877, *P* < 0.001). To construct model 2, univariate models for each of the all predictor variables were run, with CAD as the outcome variable. Those variables that were significant (*P*<0.05) in univariate logistic models were then simultaneously entered into model 2. After adjusting for multiple clinical factors include diabetes, hypertension, smoking, HR, FBG, we found AIP also could a powerful risk factor [OR = 3.619, 95%CI (2.003–6.538),*P* < 0.001] (Table 3).

**Table 3:** Univariate and multivariate logistic regression analyses for lipid parameters with CAD risk

Variables	Univariate		Model 1		Model 2	
	OR 95%*CI*	*P*	OR 95%*CI*	*P*	OR 95%*CI*	*P*
TG	1.238(1.056–1.451)	0.008	1.197(1.023–1.401)	0.025	1.222(1.038–1.438)	0.016
TC	1.151(1.004–1.320)	0.044	1.146(0.998–1.316)	0.053	1.147(0.997–1.319)	0.056
HDL	0.323(0.195–0.536)	< 0.001	0.350(0.210–0.583)	< 0.001	0.332(0.198–0.558)	< 0.001
LDL	1.279(1.086–1.506)	0.003	1.278(1.084–1.507)	0.004	1.276(1.080–1.508)	0.004
APOA-1	0.546(0.299–0.998)	0.049	0.558(0.304–1.026)	0.060	0.555(0.300–1.028)	0.061
APOB	2.009(1.182–3.414)	0.010	1.964(1.151–3.351)	0.013	1.978(1.150–3.403)	0.014
TC/HDL	1.232(1.097–1.382)	< 0.001	1.210(1.080–1.356)	0.006	1.216(1.084–1.364)	0.001
LDL/HDL	1.376(1.179–1.604)	< 0.001	1.358(1.165–1.582)	< 0.001	1.363(1.169–1.590)	< 0.001
non-HDL	1.294(1.160–1.500)	0.001	1.279(1.102–1.485)	0.001	1.286(1.105–1.496)	0.001
AI	1.232(1.097–1.382)	< 0.001	1.210(1.097–1.356)	0.001	1.216(1.084–1.364)	0.001
LCI	1.018(1.010–1.026)	< 0.001	1.017(1.008–1.025)	< 0.001	1.017(1.008–1.026)	< 0.001
API	3.685(2.081–6.532)	< 0.001	3.290(1.842–5.877)	< 0.001	3.619(2.003–6.538)	< 0.001
APOA-1/APOB	3.159(1.665–5.995)	< 0.001	3.076(1.616–5.855)	0.001	3.119(1.628–5.979)	0.001

*CI* confidence interval, *OR* odds ratio, Model 1:adjustment risk factors including hypertension, diabetes; Model 2: Those variables (hypertension, diabetes, smoking, heart rate, FBG) that were significant (*P<0.05*) in univariate models were then simultaneously entered into this multivariable model;

## Discussion

To our knowledge, there is paucity of literature on its application to postmenopausal women. Therefore, our goal was to assess the association between AIP and CAD and to determine if it might pose an independent risk factor in the postmenopausal women. A case-control study demonstrates that AIP is a usable risk factor in postmenopausal women. Among all lipoprotein markers, AIP was positively associated with Gensini score. AIP independent of smoking habit, history of diabetes mellitus and hypertension was developed as a marker of increased CAD risk that might be better than TC, LDL-C and HDL-C and other lipid concentrations.

We obtained similar AIP values (mean 0.15 ± 0.27) as Nwagha et al. [17] in their group of Nigerian postmenopausal women (mean 0.15 ± 0.35), lower than Nansseu et al. [18] Likewise, relation between AIP and known history of diabetes, hypertension and other lipid parameters which have been cited as risks factors for CVD were found [19, 20]. However, we did not found relation between AIP and BMI, SBP and uric acid, in contradiction with previous reports [6, 7, 21]. Dobiášová et al. [8] demonstrated that AIP inversely correlates with LDL-C particle size in a high diversity population composed of both high and low-risk individuals with high correlation coefficients for the first time. They suggested indeed that AIP values of − 0.3 to 0.1 may be associated with low, 0.1 to 0.24 with medium and above 0.24 with high risk of CVD. It was bolstered that the strong correlation of AIP with lipoprotein particle size may explain its high predictive value of CVD occurrence [9]. AIP was found to be a surrogate for sdLDL particles and negatively associated with LDL-C particle diameter. An increase in AIP indicates a reduction in the LDL particle diameter and a rise in the proportion of sdLDL. SdLDL particles are more prone to oxidation, to promote the formation of foam cells and LDL-C with oxidized apoprotein B is regarded as highly atherogenic characteristic. Also they can cause atherosclerosis by increasing lipid peroxidation, activating oxygen radicals, expressing adhesion molecules on endothelial cells which were linked to endothelial dysfunction [22]. HDL-C allows the use of peripheral cholesterol by transporting it to the liver. Also, it includes antioxidant enzymes such as paraoxonase [23].

This lipoprotein subfraction named AIP is well-known for its strong association with the development of CAD, all-cause mortality, and it also predicts emergency cardiovascular events [6–9, 24–27]. Dobiášová et al. [28] further proved that AIP is strongly associated with atherosclerosis and its complications by establishing an association between the AIP and coronary angiographic findings in patients with CAD. In a prospective study by Onat et al. [19] after a 7.8-year follow-up of 2676 middle-aged adults showed that AIP is a reliable biomarker for predicting CVD morbidity. To support the theory that AIP predicts the risk of development of type 2 diabetes mellitus better than traditional lipid markers, a meta-analysis of 15 studies was done [20]. Toft-Petersen et al. [25] conducted a cross-sectional study which found an association between sdLDL concentration and the presence of CAD in the elderly population. They verified that there was relation between AIP and mortality in the elderly. Significantly increased AIP and intima-media thickness of the carotid artery were found in subclinical atherosclerosis in patients on maintenance hemodialysis in Turkey [29].Similar findings of AIP as useful tool for the diagnosis and prognosis of CVD was reported in Morocco population [30]. Although this index has been used to predict the risk of atherosclerosis in hypertensive postmenopausal women in south East Nigeria [17]. However, contrary to our conclusion, Nansseu et al. [18] found AIP may not be an independent factor impacting the risk of CVD through a cross-sectional study conducted among 108 postmenopausal women in Cameroon. At the same time, atherogenic lipoproteins that have been identified, the high apoliprotein B, low apolipoprotein A-1 and the high ratio of apoB: apoA1 also affect the formation and progression of coronary atherosclerosis [31, 32]. In our study, we also find an increased ratio of apoB: apoA1 value was a highly significant risk factor of CAD independent of smoking, hypertension, and diabetes. It remains predictive after adding other confounding factors.In 2002, sdLDL was identified as a major risk factor for CAD by the National Cholesterol Education Program, and the program recommended detection of sdLDL [33]. However, all currently available methods used to detect sdLDL have limitations and suffer from the problem of high cost, which is difficult to popularize in clinical practices like the detection of TG, TC, HDL-C and LDL-C. Nevertheless, the calculation of AIP is easy and AIP effectively reflects the sdLDL level. Therefore, the use of AIP to evaluate the risk of developing atherosclerosis is feasible for the prevention and control of cardiovascular diseases in a community population.

## Limitation

In our study, we found that AIP as useful tool for the diagnosis of CAD in postmenopausal women. However, some interventions such as differences in culture, race, diet, lifestyle, demographic characteristics, laboratory tests and different postmenopausal and metabolic status could also affect AIP values. Especially the role of nutraceuticals on lipid plasma levels is very important. Nutraceuticals are not only effectively able to reduce the burden of the atherosclerosis process, coronary heart disease development, mortality and morbidity caused by CAD, it but also can be able to ameliorate human dyslipidaemia as already demonstrated in the literature by Pietro Scicchitano [34]. A variety of traditional and non-traditional nutraceuticals are used to regulate blood lipids by different molecular mechanisms. Besides, some pharmacological interventions (such as hormone replacement therapy and vitamin D supplementation) could be considered, though most of them have yielded controversial outcomes. Most importantly, oral contraceptives, or medications known to affect lipid metabolism (lipid-lowering drugs, fish oil capsules, Beta-blockers, or diuretics) should be considered. Also the retrospective nature of this paper is a limitation of its design. We made a propensity score matching case–control study to make the data between the case-control group can be compared and try our best to realize the integrity of data and eliminate the incomplete data in the process of data collection, and reflect objective facts as accurately as possible. Dyslipidaemia in our postmenopausal women is indicative of their susceptibility to atherosclerosis and other cardiovascular disorders, therefor, larger, community-based and well designed studies are warranted to better investigate the relationship between AIP and risk of CVD among postmenopausal women.

## Conclusion

The present study suggested that AIP might be a strong marker for predicting the risk of CAD in postmenopausal women.

## Abbreviations

AIP: Atherogenic index of plasma
CAD: Coronary artery disease
CAG: Coronary angiography
DM: Diabetes mellitus
EH: Essential hypertension
LVEF: Left ventricular ejection fraction
